# High value of rapid diagnostic tests to diagnose malaria within children: A systematic review and meta-analysis

**DOI:** 10.7189/jogh.10.010411

**Published:** 2020-06

**Authors:** Wenjun Zhu, XiaoXiao Ling, Wenru Shang, Yanqiu Du, Jinyu Liu, Yuanyuan Cao, Mengmeng Yang, Guoding Zhu, Jun Cao, Jiayan Huang

**Affiliations:** 1 Key Lab of Health Technology Assessment, National Health Commission; School of Public Health, Fudan University, Shanghai, China; 2 Department of Statistical Science, University College London, London, UK; 3 National Health Commission Key Laboratory of Parasitic Disease Control and Prevention, Jiangsu Provincial Key Laboratory on Parasite and Vector Control Technology, Jiangsu Institute of Parasitic Diseases, Wuxi, Jiangsu, China; 4 Center for Global Health, School of Public Health, Nanjing Medical University, Nanjing, Jiangsu, China; 5 Public Health Research Center, Jiangnan University, Wuxi, Jiangsu, China

## Abstract

**Background:**

Children aged under five years accounted for 61% of all malaria deaths worldwide in 2017, and quicker differential diagnosis of malaria fever is vital for them. Rapid diagnostic tests (RDTs) are strips to detect

**Plasmodium:**

specific antigens promptly and are helpful in resource-limited areas. Thus, our aim is to assess the diagnostic accuracy of RDTs for malaria in children against the gold standard.

**Methods:**

MEDLINE, Web of Science, EMBASE, Cochrane Library, the China National Knowledge Infrastructure, Wanfang, and Sinomed databases were systematically searched on August 23, 2019. Studies that compared RDTs with microscopy or polymerase chain reaction in malaria diagnoses for children were eligible. Relevant data were extracted. The quality of studies was evaluated using the revised Quality Assessment of Diagnostic Accuracy Studies instrument. Meta-analyses were carried out to calculate the pooled estimates and 95% confidence intervals of sensitivity and specificity.

**Results:**

51 articles were included. For diagnostic accuracy, the pooled estimates of the sensitivity and specificity of RDTs were 0.93 (95% confidence interval (CI) = 0.90, 0.95) and 0.93 (95% CI = 0.90, 0.96) respectively. Studies were highly heterogeneous, and subgroup analyses showed that the application of RDTs in high malaria transmission areas had higher sensitivity but lower specificity than those in low-to-moderate areas.

**Conclusions:**

RDTs have high accuracy for malaria diagnosis in children, and this characteristic is more prominent in high transmission areas. As they also have the advantages of rapid-detection, are easy-to-use, and can be cost-effective, it is recommended that the wider usage of RDTs should be promoted, especially in resource-limited areas. Further research is required to assess their performance in WHO South-East Asia and Americas Region.

Malaria is a preventable, curable but life-threatening disease caused by parasites including *Plasmodium falciparum* (Pf), *Plasmodium vivax* (Pv), *Plasmodium knowlesi* (Pk), *Plasmodium malariae* (Pm), and *Plasmodium ovale* (Po), of which *P. falciparum* and *P. vivax* are the most prevailing and *P. falciparum* the most deadly [1,2]. In 2017, there were around 219 million cases of malaria worldwide, and the attributable mortality was nearly 435 thousand [3]. Especially, children aged under 5-year-old accounted for 61% of all malaria deaths around the world, thus they are the most susceptible group affected by malaria [3,4]. Contrast to adults, children are more vulnerable to infectious diseases, and quicker differential diagnosis between malaria and non-malaria fever is needed for lessening death and severe cases.

According to World Health Organization (WHO), all suspected malaria cases should take a parasitological test to confirm the diagnosis using either microscopy or malaria rapid diagnostic tests (RDTs) [5]. The aim of this strategy is to reduce the unnecessary use of Artemisinin-based combination therapies (ACT) and prevent potential drug resistance [6]. In addition, it can improve the diagnosis of other non-malaria febrile diseases. Microscopic examination of blood slides is considered as the “gold standard” for malaria diagnosis, but it is time-consuming and requires well-trained personnel and adequate laboratory equipment, which is hard to maintain in most of the endemic areas [7,8]. RDTs can detect specific antigens produced by Plasmodium in individual blood, including histidine-rich protein-2 (HRP2), lactate dehydrogenase (LDH), and aldolase. HRP2 is specific for *P. falciparum*, while aldolase can be found in all species (pan-specific). LDH can be divided into three categories: Pf-specific, Pv-specific and pan-specific. Antibodies against these antigens can be combined in one type of RDTs to detect different *Plasmodium* species [9,10]. According to Bell and his colleagues, RDTs can be divided into 7 types depending on their target antigens (Appendix S1 in the **Online Supplementary Document**) [11]. Besides, pan-specific LDH only and Pv-specific LDH only tests are also available now [12]. The typical operation of RDTs is to combine a drop of finger-pricked blood and a couple of drops of buffer into RDTs cassette and wait for several minutes until the results appear on the strip. Compared to microscopy, the tests are simple to perform and interpret while providing rapid results. So, it can be used at the community level. Polymerase chain reaction (PCR) is one of nucleic acid amplification techniques, which is more sensitive than microscopy, and it can also be regarded as the “gold standard”. However, it has a higher requirement on trained technicians and standard laboratory. Thus, it does not fit the field malaria diagnosis currently and is mainly operated in epidemiological research [9,13].

Although WHO has established the diagnostic criteria, the use of parasitological tests to diagnose malaria for children was still depressed. WHO African Region accounted for 92% of all malaria cases in 2017, but according to 58 household surveys conducted in 30 sub-Saharan African countries, in 2015-2017, the median percentage of febrile children who received a diagnostic test in public health facilities was only 59% [3], which meant that there were still around two-fifths children who did not have the access to the parasitological diagnosis. Since the majority of African health facilities lack the capacity and/or device to perform microscopy [14], RDTs, which are easy to use, will be helpful to provide rapid diagnosis and avert avoidable death for children, to reach SDG 3.2 – end preventable deaths of newborns and children under-5 by 2030, and the target set by *the Global Technical Strategy for Malaria 2016-2030*, ie, at least 90% malaria incidence and mortality should be reduced by 2030.

The validity of RDTs has been approved in recently published systematic reviews [9,12,15], but all of them do not have restrictions on the age of the target population. The validity of RDTs in childhood malaria diagnosis has its own characteristics and may be different from adults. That is because the immunity towards *Plasmodium* increases with age [16], and the anti-parasite ability of children is lower than adults. It would lead to a higher parasite density of childhood malaria infection if other conditions are the same [17].

Clinical evidence has been accumulated on the operations of RDTs for childhood malaria diagnosis. However, wide disparities in their performance have been observed across studies [18-22]. These discrepancies may be attributed to different study designs, sample size, study location and reference standard used. Therefore, a systematic review was conducted to provide a comprehensive evaluation of the diagnostic accuracy and investigate the performance of RDTs against the gold standard in malaria diagnosis among children.

## METHODS

### Search strategy and selection criteria

A systematic approach was used to search the following databases: Pubmed, Web of Science, EMBASE, Cochrane Library, the China National Knowledge Infrastructure (CNKI), Wanfang Data, and Sinomed. The latter three are Chinese databases. The search strategies were outlined in Appendix S2 in the **Online Supplementary Document** and no restriction was imposed. The search was undertaken on August 23, 2019, and the references of all eligible studies were checked manually to identify extra relevant articles.

The inclusion criteria were as follows: (1) Primary studies that evaluated the diagnostic accuracy of RDTs. (2) The microscopic examination of blood smears or PCR was selected as the gold standard. (3) Participants were children. (4) Studies that reported the direct comparison results between RDTs and the gold standard. The exclusion criteria were as follows: (1) Studies that were case reports, reviews, editorials, letters, comments, and conference abstracts. (2) Studies that did not present enough information to extract or calculate the number of true-positives, false-positives, true-negatives, and false-negatives. The title and abstract of all relevant articles were read by three reviewers independently according to the inclusion and exclusion criteria during the first round of screening. Then the full texts of the eligible studies were rechecked based on the same criteria. Any disagreement between three reviewers was resolved by discussion.

### Data extraction and quality assessment

The following data were independently extracted by three reviewers from eligible studies using Microsoft Excel 2016 (Microsoft Inc, Seattle WA, USA): (1) study characteristics: journal, publication year, first author and his/her institution, study period, study setting, and study design. (2) participants’ characteristics: the inclusion and exclusion criteria, sample size, the number of malaria cases, the age range and sex distribution of participants, and the parasite density of *Plasmodium*. (3) RDTs characteristics: commercial brand, and specific *Plasmodium* species and antigens detected. (4) RDTs performance: the reference standard, and the number of true-positives, false-positives, true-negatives, and false-negatives. Any discrepancy between three reviewers was resolved by discussion. If only a subset of participants met the selection criteria, data were extracted only for the subgroup.

The revised Quality Assessment of Diagnostic Accuracy Studies (QUADAS-2) tool was used to assess the methodological quality of the eligible studies [23], as recommended by the Cochrane Collaboration. The tool has four domains: patient selection, index test, reference standard, and flow and timing. Each domain was scored as “high/low risk of bias” and “high/low applicability concern”, except for the last one which only contains the risk of bias section. If insufficient data were reported, the corresponding section would be classified as “unclear”. Reviewers assessed the quality of studies independently using Review Manager 5.3 and discussed the inconsistencies. The criteria for each section are listed in Appendix S3 in the **Online Supplementary Document**.

### Statistical analysis

We estimated the sensitivity and specificity of each study with 95% confidence intervals (CI) and presented the results in forest plots. Then we used the *Midas* module in Stata 12.1 (StataCorp LLC, Texas, USA) to calculate the pooled estimates of the sensitivity, specificity, positive likelihood ratio, negative likelihood ratio, and diagnostic odds ratio. Midas is a comprehensive program for undertaking meta-analysis of diagnostic test accuracy in Stata. Its primary data synthesis is based on the bivariate mixed-effects regression framework. A hierarchical summary receiver operating characteristic curve (HSROC) was fitted and funnel plots were presented respectively to show the comprehensive diagnostic value of RDTs and the potential publication bias among eligible studies. Compared with the summary receiver operating characteristic (SROC) model, the HSROC model allows more between and within-study variability [24]. It was adopted as studies included are expected to show considerable heterogeneity in diagnostic accuracy [25]. We also performed the Q test to assess the heterogeneity among the included studies. The extent of heterogeneity was quantified by I^2^ measure [26]. If the heterogeneity was significant (I^2^>50%), we used Meta-disc 1.4.0 software (the Unit of Clinical Biostatistics team of the Ramón y Cajal Hospital, Madrid, Spain) to explore whether a threshold effect existed. Furthermore, meta-regression was conducted to investigate the potential sources of heterogeneity. Covariates included local malaria transmission type, study design, sampling method, reference standard, sample size, geographic location, blinding status, RDTs type, and target antigens. In the regression, the accuracy measure was relative diagnostic odds ratio (RDOR). The coefficients of covariates indicated the change in the diagnostic performance of the RDTs under each study per unit increase in the covariates. In other words, *P* < 0.05 represented that the corresponding covariates were the major sources of heterogeneity. Subgroup analyses based on the sources of heterogeneity were subsequently conducted by Stata (Stata Corp, College Station, TX, USA).

For meta-regression and subgroup analyses, transmission type was divided into four categories: high, low-to-moderate, mixed and unclear. The transmission type was classified as “high” if the authors described it as “hyperendemic”, “perennial”, “holoendemic” or “high”; “low-to-moderate” when it was “mesoendemic”, “sporadic”, “moderate” or “low”; “mixed” if it was described as “seasonal” or when multiple sites of different transmission types were included; “unclear” if the transmission type was not mentioned.

## RESULTS

### Results of the search

**Figure 1:**
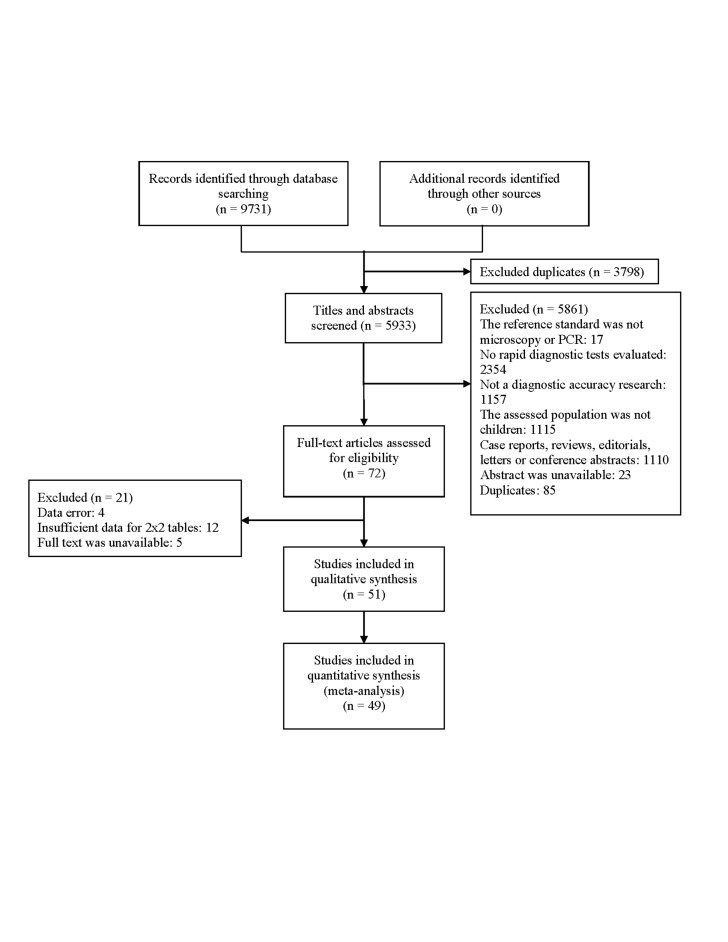
Flowchart of the selection procedure. PCR – polymerase chain reaction.

A total of 9731 relevant articles were identified. After removing duplicates, 5933 articles were selected. 5861 articles were excluded based on the criteria. The full texts of 72 articles were evaluated and 51 of them were eventually included. Among them, the most common reason for exclusion was the lack of data for a 2x2 table. The detailed process for selection is shown in **Figure 1**.

Out of 51 studies included in the review, two evaluated the validity of RDTs when they were used to monitor the effects of ACT treatment, and the others assessed the diagnostic capacity of RDTs. Since HRP2 will be cleared slowly from bloodstream if the treatment of *P. falciparum* is successful and it can contribute to a higher false-positive rate of RDTs [27], our analyses were grouped into two parts according to the usage time of RDTs (before or after ACT treatment). There were 9 studies that adopted multiple types of RDTs and/or reference standards: 6 studies evaluated 2 types of RDTs [28-33], 2 studies evaluated 3 types of RDTs [18,34], and 2 studies selected two different types of reference standards [32,35]. Particularly, in one study, two staffs read the RDTs strips respectively, so there were two different results for each type of RDTs [29]. As enrolled children were asked to retest blood samples during the follow-up regularly, the studies that assessed the RDTs capacity for monitoring the effect of ACT treatment all have multiple test evaluations. As a result, we had 82 test evaluations reporting a total of 57 312 test results. Among them, 34.45% of tests (19 746) showed the positive result against the reference standard. Most of the studies included were conducted in Africa (n = 47), and the rest of them happened in Asia (three in India and one in Pakistan). The detailed characteristics of the included studies were summarized in **Table 1**.

**Table 1:** Detailed characteristics of included studies

	Study	Country*	Transmission type*	Study design	Sample size	Children age range*	Sex (M/F)*	Parasite density range (parasites/μl)*	RDTs target antigens*	Reference standard*
1	Singh, 2002 [36]	India	Mesoendemic	Cross-sectional study	573	5Y-14Y	/	78-6360	HRP2	Microscopy
2	Mtove, 2011 [19]	Tanzania	Perennial	Prospective cohort	965	3M-59M	/	/	HRP2	Microscopy
3	Shaikh, 2013 [37]	Pakistan	Moderate	Cross-sectional study	400	2M-5Y	140/260	/	/	Microscopy
4	Keating, 2009 [20]	Zambia	/	Cross-sectional study	618	0M-59M	324/294	6-678	HRP2	Microscopy
5	Owusu, 2018 [38]	Ghana	Perennial	Cross-sectional study	401	≤5Y	98/303	/	HRP2	PCR
6	Kashosi, 2017 [39]	DR Congo	Sporadic & seasonal	Cross-sectional study	460	6M-59M	235/225	/	HRP2 & pan-specific LDH	Microscopy
7	Ayeh, 2011 [40]	Ghana	/	Cross-sectional study	200	4D-5Y	/	/	Pf-specific LDH & pan-specific LDH	Microscopy
8	Kiemde, 2017 [41]	Burkina Faso	/	Cross-sectional study	684	<5Y	369/314	/	HRP2	Microscopy
9	Baiden, 2012 [42]	Ghana	/	Prospective cohort	436	3M-60M	236/200	31-1 518 575	HRP2	Microscopy
10	Gerstl, 2010 [28]	Sierra Leone	Hyperendemic	Prospective cohort	343	2M-58M	177/166	1-2 136 000	Pf-specific LDH & pan-specific LDH; HRP2	Microscopy
11	Premji, 1994 [43]	Tanzania	/	Cross-sectional study	380	≤42M	/	/	HRP2	Microscopy
12	Singh, 2001 [44]	India	/	Cross-sectional study	191	2Y-10Y	/	/	HRP2	Microscopy
13	Nkrumah, 2011 [6]	Ghana	/	Cross-sectional study	263	3Y-16Y	147/116	50-8320	HRP2 & aldolase	Microscopy
14	Hopkins, 2007 [29]	Uganda	/	Prospective cohort	918	1.5Y-11.5Y	/	/	HRP2; only pan-specific LDH	Microscopy
15	Adesanmi, 2011 [45]	Nigeria	/	Cross-sectional study	380	6M-59M	/	/	HRP2	Microscopy
16	Bojang, 1999 [46]	Gambia	High	Cross-sectional study	139	/	/	/	HRP2	Microscopy
17	Hendriksen, 2011 [30]	Tanzania; Mozambique	High; Lower	Cross-sectional study	1898	/	990/908	/	HRP2; Pf-specific LDH & pan-specific LDH	Microscopy
18	Rabiu, 2013 [21]	Nigeria	/	Cross-sectional study	209	6M-12Y	106/103	40-203 883	HRP2	Microscopy
19	Mens, 2007 [34]	Kenya; Tanzania	High; Mesoendemic	Cross-sectional study	338	6M-12Y	191/147	/	Pf-specific LDH & pan-specific LDH; HRP2; HRP2 & pan-specific LDH	Microscopy
20	Kamugisha, 2008 [47]	Tanzania	/	Cross-sectional study	301	/	163/138	/	HRP2	Microscopy
21	Valéa, 2009 [48]	Burkina Faso	Hyperendemic	Cross-sectional study	464	6M–59M	/	40-234 280	Pf-specific LDH & pan-specific LDH	Microscopy
22	Hawkes, 2014 [49]	Uganda	/	Cross-sectional study	2000	2M-5Y	1114/886	/	HRP2 & pan-specific LDH	Microscopy
23	Oyeyemi, 2016 [22]	Nigeria	/	Cross-sectional study	200	/	102/98	/	HRP2	Microscopy
24	Mahende, 2016 [50]	Tanzania	Low to moderate	Cross-sectional study	867	2M-59M	459/408	/	HRP2	PCR
25	Ilombe, 2014 [51]	DR Congo	/	Cross-sectional study	872	/	469/403	/	HRP2 & pan-specific LDH	Microscopy
26	Ajumobi, 2015 [52]	Nigeria	Mesoendemic; Seasonal	Cross-sectional study	300	6–59M	163/132	/	HRP2	Microscopy
27	Sotimehin, 2007 [53]	Nigeria	/	Cross-sectional study	205	0D–3D	/	17-2940	Pf-specific LDH & pan-specific LDH	Microscopy
28	Tarimo, 2015 [54]	Tanzania	Intensive perennial	Cross-sectional study	474	0-59M	246/220	/	HRP2 & pan-specific LDH	Microscopy
29	Ratsimbasoa, 2012 [35]	Madagascar	High; Low	Cross-sectional study	543	2-59M	-	48-82 000	HRP2 & pan-specific LDH	PCR; Microscopy
30	Wanji, 2008 [55]	Cameroon	Hyperendemic	Cross-sectional study	186	4Y-16Y	88/98	/	only pan-specific LDH	Microscopy
31	Tiono, 2013 [56]	Burkina Faso	Seasonality	Cross-sectional study	525	6M-59M	248/277	/	HRP2	Microscopy
32	Swarthout, 2007 [57]	DR Congo	High; Seasonal	Cross-sectional study	358	6M-59M	/	/	HRP2	Microscopy
33	Samadoulougou, 2014 [58]	Burkina Faso	/	Cross-sectional study	6260	6M-59M	3181/3079	/	HRP2	Microscopy
34	Grandesso, 2016 [18]	Uganda	Low; High	Prospective cohort	5262	/	2711/2551	/	HRP2; HRP2; only pan-specific LDH	Microscopy
35	Venkatesh, 2007[59]	India	/	Cross-sectional study	149	≤12Y	/	/	HRP2	Microscopy
36	Bouyou, 2013 [31]	Gabon	Perennial	Cross-sectional study	386	≤10Y	/	/	HRP2 & aldolase; HRP2	Microscopy
37	Aydin, 2013 [60]	Tanzania	Moderately high	Prospective cohort	53	10M-59M	35/18	2000-250 000	HRP2	PCR
38	Houze, 2009 [33]	Benin	High; Seasonal	Prospective cohort	205	6M-59M	/	1000-525 000	HRP2; Pf-specific LDH & pan-specific LDH	Microscopy
39	Adebisi, 2018 [61]	Nigeria	..	Cross-sectional study	370	<5Y	211/159	/	HRP2	Microscopy
40	Al, 2019 [62]	Uganda	Mixed	Cross-sectional study	247	..	124/123	/	HRP2 & pan-specific LDH	PCR
41	Bloch, 2018 [63]	Tanzania	..	Cross-sectional study	1049	1M-59M	527/503	/	/	Microscopy
42	Dada, 2018 [64]	Nigeria	holoendemic	Cross-sectional study	102	7M-17Y	58/44	/	HRP2	Microscopy
43	Nkefou, 2018 [65]	Cameroon	..	Cross-sectional study	249	6M-15Y	134/115	/	HRP2 & pan-specific LDH	Microscopy
44	Hofmann, 2019 [66]	Tanzania	Low	Cross-sectional study	3192	2M-59M	1766/1426	/	HRP2	PCR
45	Iwuafor, 2018 [67]	Nigeria	High	Cross-sectional study	270	1M-59M	/	/	HRP2	Microscopy
46	Kiemde, 2019 [68]	Burkina Faso	Seasonal	Cross-sectional study	407	≤5Y	231/176	/	HRP2 & pan-specific LDH	Microscopy
47	Kiemde, 2018 [69]	Burkina Faso	Seasonal	Cross-sectional study	407	≤5Y	231/176	32–58 625	HRP2	Microscopy
48	Kitutu, 2018 [70]	Uganda	Low	Cross-sectional study	212	2M-60M	106/95	/	HRP2	PCR
49	Peprah, 2019 [71]	Tanzania	Mixed	Cross-sectional study	819	0Y-15Y	432/387	/	HRP2	Microscopy
50	Quakyi, 2018 [32]	Ghana	Mixed	Prospective cohort	260	<5Y	131/129	300-99 500	HRP2; HRP2 & pan-specific LDH	Microscopy; PCR
51	Teh, 2019 [72]	Cameroon	perennial	Cross-sectional study	491	6M-14Y	218/273	70-1162	HRP2	Microscopy

RDTs – malaria rapid diagnostic tests, Y – years, M – months, D – days, M – male, F – female, HRP2 – histidine-rich protein-2, LDH – lactate dehydrogenase, Pf – *Plasmodium falciparum*, Pan - all *Plasmodium* species, PCR – polymerase chain reaction, DR Congo – The Democratic Republic of the Congo, / – missing data, & – and

*Semicolons separate different test evaluations.

### The methodological quality of the included studies

**Figure 2:**
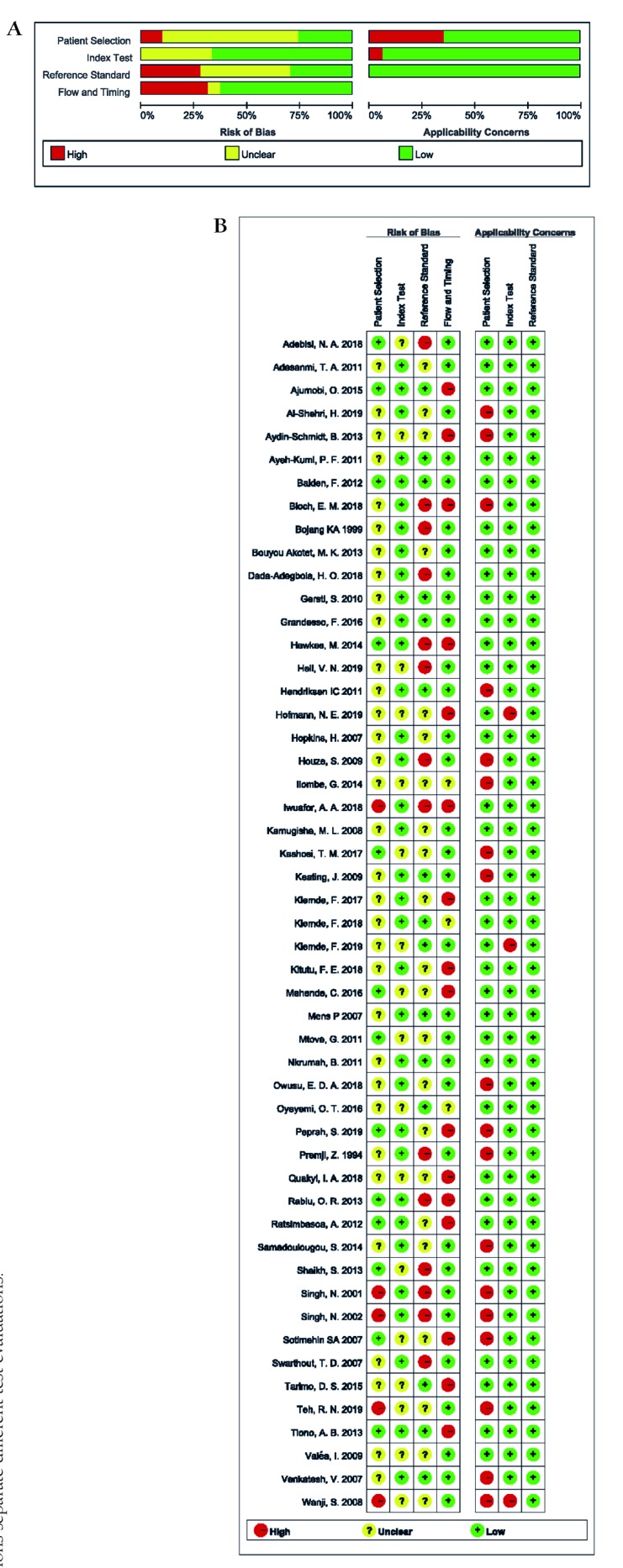
Methodological quality assessment of studies included in the review. **Panel A.** Overall quality of studies included in the review. **Panel B.** Detailed quality of studies included in the review.

The overall methodological quality of studies included was relatively high, as the scores of risk of bias and applicability concerns in the four domains were mainly low. Most studies (n = 41) enrolled consecutive or random sample of patients. None of them were case-control studies. 17 studies adopted double-blind method, and 1 study was single-blind, but the rest did not supply sufficient information. 6 studies selected PCR as the reference standard, 43 studies took microscopy as the reference standard, and 2 studies chose both. 31.37% (16/51) of the eligible studies were scored to have a high risk of bias in the flow and timing domain since they did not include all patients in the analysis. 35.29%(18/51) of included studies had a high applicability concern in patient selection domain because of their unrepresentative samples. The results of the methodological quality were shown in **Figure 2**, Panel A and Panel B.

### Diagnostic accuracy of RDTs

There were 63 test evaluations that focused on the diagnostic accuracy of RDTs [6,18-22,28-32,34-59,61-72]. 59 were conducted in Africa and 4 in Asia. 55 tests selected microscopy as the reference standard and 8 chose PCR. The median sample size was 400 (range: 102 - 6260). 14 tests evaluated the performance of RDTs in a high malaria transmission setting, 6 in low-to-moderate and 20 in mixed.

**Figure 3:**
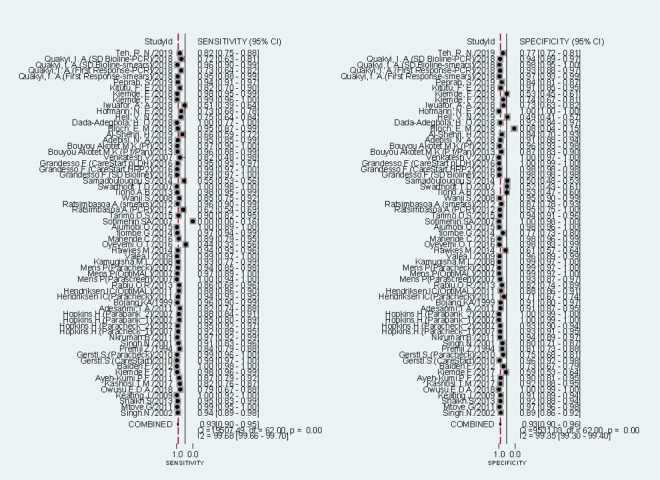
Forest plots of sensitivity and specificity of RDTs.

**Figure 4:**
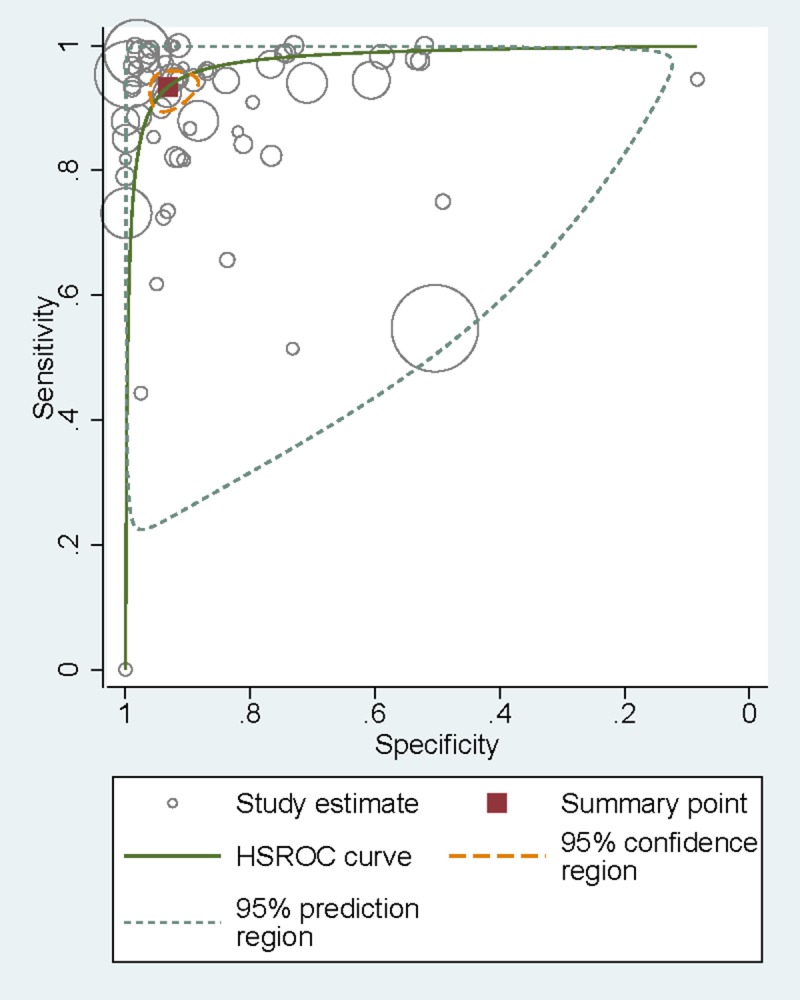
HSROC curve of sensitivity vs specificity of RDTs. HSROC – hierarchical summary receiver operating characteristic curve.

**Figure 5:**
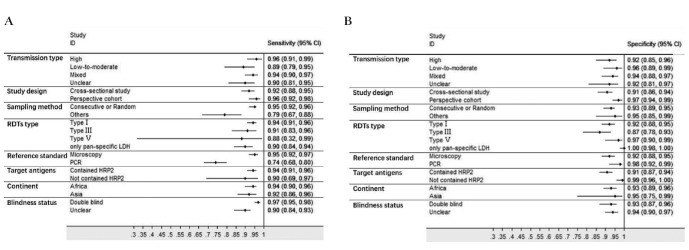
The effect of each categorical variables on the accuracy of RDTs. **Panel A.** The effect of each categorical variables on the sensitivity of RDTs. Type II, type unclear, and single-blind groups did not have enough test evaluations to perform meta-analysis. HRP2 – histidine-rich protein-2. LDH = lactate dehydrogenase. Pan = all Plasmodium species. PCR – polymerase chain reaction. **Panel B.** The effect of each categorical variables on the specificity of RDTs. Type II, type unclear, and single-blind groups did not have enough test evaluations to perform meta-analysis. HRP2 – histidine-rich protein-2. LDH – lactate dehydrogenase. Pan – all Plasmodium species. PCR – polymerase chain reaction.

**Figure 6:**
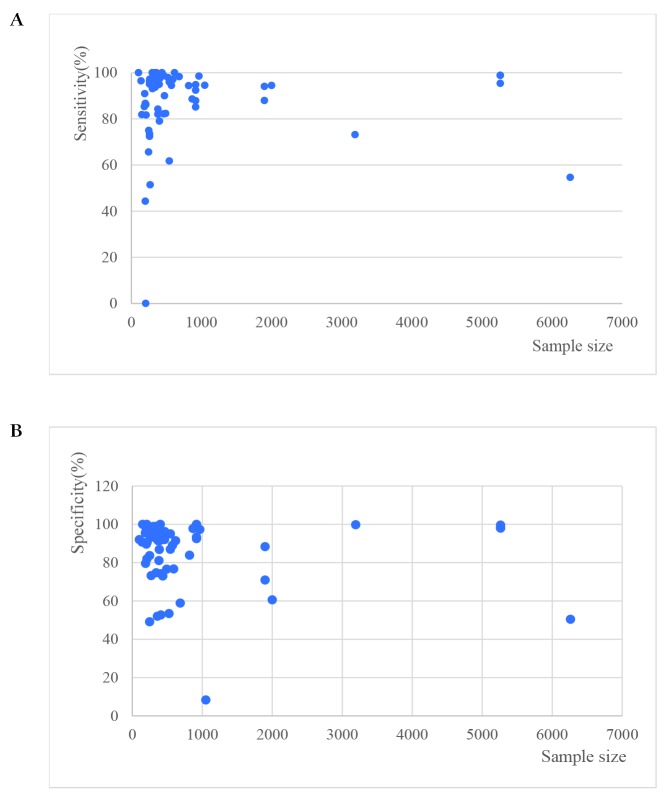
The effect of sample size on the accuracy of RDTs. **Panel A.** The effect of sample size on the sensitivity of RDTs. **Panel B.** The effect of sample size on the specificity of RDTs.

Sensitivities of tests ranged from 0.00 to 1.00, and specificities from 0.08 to 1.00 (**Figure 3**). The pooled summary of sensitivity and specificity (95% CI) of RDTs were 0.93 (0.90-0.95) and 0.93 (0.90-0.96) respectively. The pooled estimates for the positive likelihood ratio, negative likelihood ratio, and diagnostic odds ratio (95% CI) were 13.67 (8.94-20.90), 0.07 (0.05-0.10), and 192.67 (111.46-333.06) respectively. HSROC curve summarized the sensitivity and specificity of RDTs in **Figure 4**. The area under the curve is close to 100%, indicating that the performance of RDTs was satisfactory. High heterogeneity was observed between studies (Cochrane’s Q = 2182.22, I^2^ = 100.00, *P* < 0.001), thus we explored its source through the threshold effect analysis and meta-regression. The results suggested that there was no threshold effect between studies (*P* = 0.06), while transmission type, sampling method and study design were the major sources of heterogeneity (*P* < 0.05). The results of the meta-regression were shown in **Table 2**. In addition, the effect of each variable on the accuracy of RDTs was presented by forest plots if the variable was categorical (**Figure 5**, Panel A and Panel B), and by scatter plots if it was continuous (**Figure 6**, Panel A and Panel B).

**Table 2:** Meta-regression analysis of diagnostic accuracy

	Coefficient	Standard error	*P*-value	RDOR	95% CI
Cte	7.83	2.23	0.00	NA	NA
S	-0.40	0.12	0.00	NA	NA
Transmission type	-0.46	0.16	0.00	0.63	0.46-0.86
Study design	1.18	0.58	0.05	3.24	1.01-10.45
Sampling method	-2.16	0.83	0.01	0.12	0.02-0.61
RDTs type	-0.23	0.25	0.38	0.80	0.48-1.32
Reference standard	-1.32	0.74	0.08	0.27	0.06-1.19
HRP2 based or not	0.49	0.88	0.58	1.63	0.28-9.51
Sample size	0.00	0.00	0.05	1.00	1.00-1.00
Continent	1.57	1.11	0.16	4.82	0.52-44.32
Blinding status	-0.33	0.28	0.25	0.72	0.41-1.27

RDTs – malaria rapid diagnostic tests, HRP2 – histidine-rich protein-2, NA – not applicable, CI – confidence interval, RDOR – relative diagnostic odds ratio, Cte – constant term in the equation, S – a measure of threshold

**Figure 7:**
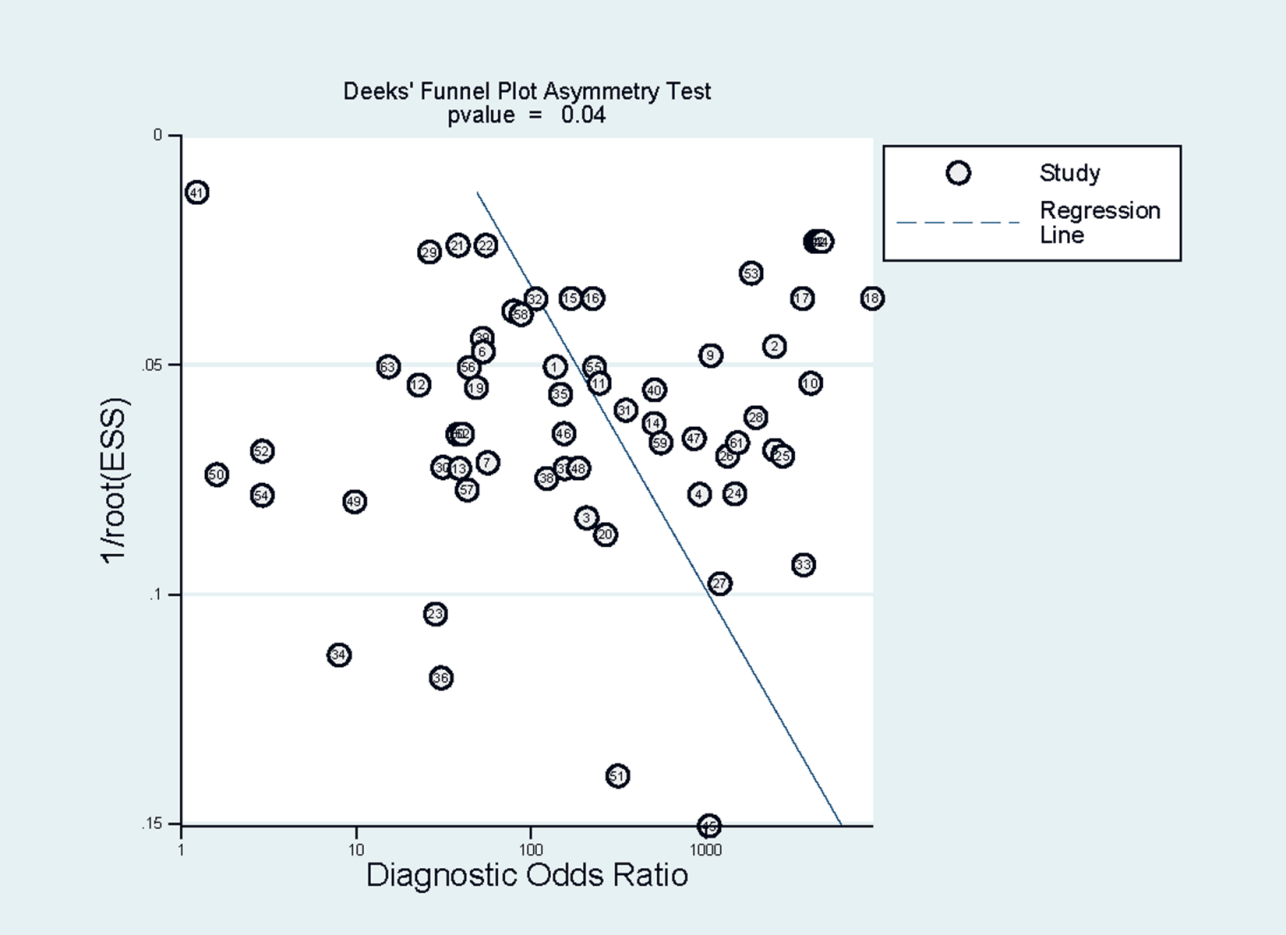
Publication bias of studies included in the review.

Subgroup analyses were conducted and the results were shown in **Table 3**. In brief, RDTs conducted in high malaria transmission areas had higher sensitivity but lower specificity compared to low-to-moderate areas. The studies with consecutive or random sample of patients presented higher sensitivity than others. Both sensitivity and specificity estimated by prospective cohort studies appeared to be higher in comparison with cross-sectional studies. A funnel plot was presented in **Figure 7**. It demonstrated the existence of publication bias (*P* = 0.04), and it was found that studies with high accuracy results tended to be published.

**Table 3:** Subgroup analysis of diagnostic accuracy

	Category	No. of tests	Sensitivity (95% CI)	Specificity (95% CI)	Positive likelihood ratio (95% CI)	Negative likelihood ratio (95% CI)	Diagnostic odds ratio (95% CI)
Transmission type	High	14	0.96 (0.91-0.99)	0.92 (0.85-0.96)	12.7 (6.4-25.3)	0.04 (0.02-0.10)	323 (103-1005)
	Low-to-moderate	6	0.89 (0.79-0.95)	0.96 (0.89-0.99)	25.3 (7.7-83.0)	0.11 (0.06-0.22)	223 (66-759)
	Mixed	20	0.94 (0.90-0.97)	0.94 (0.88-0.97)	15.0 (8.0-28.2)	0.06 (0.03-0.10)	254 (104-618)
	Unclear	23	0.90 (0.81-0.95)	0.92 (0.81-0.97)	11.5 (4.6-28.5)	0.11 (0.06-0.20)	107 (39-290)
Sampling method	Consecutive or random	54	0.95 (0.92-0.96)	0.93 (0.89-0.95)	13.0 (8.3-20.5)	0.06 (0.04-0.09)	226 (125-409)
	Others	9	0.79 (0.67-0.88)	0.95 (0.85-0.99)	16.8 (5.0-56.5)	0.22 (0.13-0.36)	78 (20-294)
Study design	Cross-sectional study	48	0.92 (0.88-0.95)	0.91 (0.86-0.94)	10.2 (6.4-16.1)	0.09 (0.06-0.13)	115 (64-207)
	Prospective cohort	15	0.96 (0.92-0.98)	0.97 (0.94-0.99)	34.4 (15.6-75.9)	0.04 (0.02-0.08)	897 (359-2242)
HRP2 based or not	Contained HRP2	51	0.94 (0.91-0.96)	0.91 (0.87-0.94)	10.9 (7.4-16.0)	0.07 (0.05-0.10)	158 (90-278)
	Not contained HRP2	10	0.90 (0.69-0.97)	0.99 (0.96-1.00)	85.9 (21.9-336.7)	0.10 (0.03-0.35)	847 (194-3704)
	Unclear	2	/	/	/	/	/

CI – confidence interval, HRP2 – histidine-rich protein-2, /– did not have enough test evaluations to perform the meta-analysis

Many studies discussed the diagnostic value of HRP2 based RDTs vs LDH based RDTs [73,74], and the problem about which type of RDTs is better still exists. Although target antigens were not the major sources of heterogeneity, a subgroup analysis was performed based on it. Results showed that HRP2 based RDTs had higher sensitivity but lower specificity than RDTs that did not contain HRP2 (**Table 3**). But there was no statistically significant difference.

### RDTs capacity for monitoring the effect of ACT treatment

19 tests evaluated RDTs’ capacity of monitoring the effect of ACT treatment [33,60]. All were conducted in Africa. Fourteen tests took microscopy as the reference standard and five took PCR. Since the days after initial treatment is an important factor affecting the accuracy of RDTs, we analyzed the results based on this framework. Consequently, after categorizing based on the follow-up period, there was no more than 3 tests within each category, and thus we could not perform a meta-analysis (To perform *Midas*, a minimum of four 2 × 2 tables is required). In the lack of statistical pooling, we presented the findings in a narrative table (**Table 4**). In short, the specificity of HRP2 based RDTs increased with the follow-up period. And at early stages after the initial treatment, the specificity of Pf-LDH based RDTs was much higher than HRP2 based RDTs.

**Table 4:** RDTs capacity for monitoring the effect of ACT treatment classified by days of follow-up

	RDTs target antigens	Days after initial treatment	TP	TN	FP	FN	Sensitivity (%)	Specificity (%)
Aydin-Schmidt, 2013 [60]	HRP2	Day 14	3	8	32	0	100.00	20.00
		Day 21	1	15	27	0	100.00	35.71
		Day 28	1	24	18	0	100.00	57.14
		Day 35	1	32	10	0	100.00	76.19
		Day 42	2	38	3	0	100.00	92.68
Houze, 2009 [33]	HRP2	Day 3	35	28	134	0	100.00	17.28
		Day 7	6	49	115	1	85.71	29.88
		Day 14	6	87	69	1	85.71	55.77
		Day 21	14	104	38	2	87.50	73.24
		Day 28	13	92	63	2	86.67	59.35
		Day 35	9	79	4	2	81.82	95.18
		Day 42	2	73	4	1	66.67	94.81
Houze, 2009 [33]	Pf-specific LDH & pan-specific LDH	Day 3	28	141	21	7	80.00	87.04
		Day 7	5	151	13	2	71.43	92.07
		Day 14	5	150	6	2	71.43	96.15
		Day 21	16	137	5	0	100.00	96.48
		Day 28	13	122	3	2	86.67	97.60
		Day 35	9	81	2	2	81.82	97.59
		Day 42	2	77	0	1	66.67	100.00

RDTs – malaria rapid diagnostic tests, HRP2 – histidine-rich protein-2, LDH – lactate dehydrogenase, Pf – *Plasmodium falciparum*, Pan - all *Plasmodium* species, TP – true-positives, TN – true-negatives, FP – false-positives, FN – false-negatives

## DISCUSSION

Our results demonstrated that RDTs had relatively high sensitivity and specificity for malaria diagnosis in children and all the findings were reported based on the PRISMA Checklist (Appendix S4 in the **Online Supplementary Document**) [75]. Since there is no previous systematic review focused on children, the results were compared with those of the whole population. Abba’s research found that the sensitivity of RDT varied between 0.915 and 0.995, while its specificity ranged from 0.906 and 0.987[9], which is comparable to our results. Moreover, in high transmission areas, the sensitivity and specificity were higher among children (0.96 and 0.92, respectively) than the whole population (0.937 and 0.896, respectively) [9]. This may relate to the fact that because adults have greater immune status than children, adult patients with malaria is more likely to have lower parasite density [60,76], and it could be difficult for RDTs to detect the low concentration of antigens among them. This characteristic makes RDTs more suitable for childhood malaria detection in high transmission areas. Another research conducted by Li calculated the accuracy of HRP2 based RDTs [15]. Comparatively, it had lower sensitivity (0.94 vs 0.96, respectively) but higher specificity (0.91 vs 0.86, respectively) in children than in adults.

Besides high diagnostic accuracy, RDTs also have the advantages of rapid detection and are easy-to-use, making it feasible to utilize it at primary health care centers. These advantages can be particularly important for *P. falciparum* detection, as it can progress rapidly from an uncomplicated febrile illness to potentially deadly disease [77]. Furthermore, compared to microscopy or PCR, the diagnostic cost of RDTs is relatively low, with a low cost of RDT strips and the training fees for laboratory staff. A few studies have been undertaken to evaluate the economic value of RDTs and they demonstrated that in comparison with microscopy, RDTs are more cost-effective if the whole treatment course have been taken into account [78-80]. Therefore, as most of the malaria-endemic areas have limited resources, RDTs is of high value to be used there. For instance, in a large proportion of African lower-level health facilities, technical expertise and microscopy were not available for children [81]. Likewise, almost half of the suspected malaria patients seek care in the private sector in Africa [82], which could be even less equipped.

Considering the endemicity of malaria, RDTs performed in high transmission areas had higher sensitivity but lower specificity than those conducted in low-to-moderate areas. This may be because low-density infection represents a significant proportion of malaria infections among children in low-transmission settings [83,84], leading to a higher false-negative rate. Furthermore, for HRP2 based RDTs, the remaining HRP2 antigen will last for several weeks in peripheral blood after a successful treatment, leading to false-positive results [27,57]. This is more common in high transmission areas since the children there may be infected with *P. falciparum* several times across their lives [28,60].

Though we did not impose any restriction on the country or region, only four studies conducted in Asia were included, and the rest of them were all performed in Africa. However, each endemic area has its own epidemiological characteristics, and the evidence of Africa cannot verify the applicability of RDTs in other areas. For instance, in the WHO South-East Asia Region, where the incidence rate was 7.0 per 1000 population at risk in 2017, both *P. falciparum* and *P. vivax* were dominant parasites [3,85]. *P. knowlesi* infection was also widely distributed there [86]. Meanwhile, most countries are confronted with the problem of limited resource. For example, India carries a high proportion of disease burden, however, microscopies were not accessible for suspected children in poor, remote villages [36,44]. Another endemic area is the WHO Americas Region, where the incidence rate was 7.3 per 1000 population at risk in 2017 [3]. Evidence demonstrated that a large proportion of *P. falciparum* lacked pfhrp2 or pfhrp3 or both genes there [87], which may lead to invalidity of HRP2 based RDTs. Therefore, corresponding research conducted in these areas is urgently needed.

There are two limitations to be considered in this study. First, since the parasite density of patients is a critical factor for the sensitivity of RDTs, we intended to perform a subgroup analysis. However, almost half of the included studies did not report the geometric mean parasite densities of patients, so linear regression could not be performed. Furthermore, it seemed that there was no widely-recognized standard for the classification of *Plasmodium* parasite density, and most of the studies classified it differently. Also, because none of the studies provided individual-level data, we could not classify the parasite density by ourselves. As a result, we could not add this factor into meta-regression and subgroup analyses, which might introduce bias. Second, our findings may be more transferable to Africa as most of the included studies were conducted there.

## CONCLUSIONS

This systematic review shows the high value of RDTs in malaria diagnosis among children. Considering current prevalence of malaria, RDTs should be a suitable diagnostic test for children, especially in resource-limited areas.

## Additional material

Online Supplementary Document
