# Supplementary material for: High value of rapid diagnostic tests to diagnose malaria within children: A systematic review and meta-analysis
Source: J Glob Health. 2020 Apr 30;10(1):010411. doi: 10.7189/jogh.10.010411 (PMC7182354; doi:10.7189/jogh.10.010411)
Supplement: Online Supplementary Document [file jogh-10-010411-s001.pdf]

Appendix S1: RDTs type

RDTs tpye	Target antigens	Possible results
Ⅰ	HRP2	No Pf; Pf; invalid
Ⅱ	HRP2 ; aldolase	No malaria; Pf or mixed; Pv, Po and/or Pm; invalid
Ⅲ	HRP2; pan-specific LDH	No malaria; Pf or mixed; Pv, Po and/or Pm; invalid
Ⅳ	Pf-specific LDH; pan-specific LDH	No malaria; Pf or mixed; invalid
Ⅴ	Pf-specific LDH; Pv-specific LDH	No malaria; Pf; Pv; Pf and Pv; invalid
Ⅵ	HRP2; pan-specific LDH; Pv-specific LDH	No malaria; Pf and Pv +/–Po and/or Pm; Pf +/– Po and/or Pm; Pv +/– Po and/or Pm; Po and/or Pm; invalid
Ⅶ	aldolase	No malaria; Pf, Pv, Po and/or Pm; invalid

Appendix S2: Search strategy

Search set	MEDLINE	Items found
#1	exp malaria/	
#2	exp plasmodium/	
#3	malaria.ab,kw,ti.	
#4	plasmodium.ab,kw,ti.	
#5	1 or 2 or 3 or 4	
#6	exp immunochromatography/	
#7	exp chromatography/	
#8	exp immunoassay/	
#9	(rdt or 'rapid diagnostic test' 'rapid diagnos* test*' or Dipstick* or 'Rapid diagnos* device*').ab,kw,ti.	
#10	('immunoaffinity chromatography' or 'immunochromatograph*' or 'antigen detection method*' or 'rapid malaria antigen test*' or 'chromatography').ab,kw,ti.	
#11	('enzyme linked immunosorbent assay' or 'rapid test' or 'reagent strip*' or 'agglutination test' or immunoassay* or 'immuno assay*').ab,kw,ti.	
#12	6 or 7 or 8 or 9 or 10 or 11	
#13	exp sensitivity/	
#14	exp specificity/	
#15	(sensitivity or specificity or "reference value" or "false positive*" or "false negative*" or "detection rate" or "diagnostic accuracy" or "laboratory diagnosis" or "diagnostic accuracy" or "diagnostic error" or "performance").ab,kw,ti.	
#16	13 or 14 or 15	
#17	5 and 12 and 16	1729
	Web of Science	
#1	TS=(plasmodium OR malaria) index=SCI-EXPANDED, SSCI	
#2	TS=(RDT OR 'rapid diagnos* test*' OR dipstick* OR 'rapid diagnos* device*' OR immunochromatograph* OR 'chromatography' OR 'antigen detection method*' OR 'rapidmalaria antigen test*' OR immunoassay OR 'enzyme linked immunosorbent assay' OR 'rapid test' OR 'reagent strip*' OR 'agglutination test' OR 'immunoassay' OR 'rapid test' OR 'reagent strip*' OR 'agglutination test' OR 'immunoassay' OR 'OptiMal®' OR	

	'ParaSight-F' OR 'Binax NOW®' OR 'ICT Malaria' OR "CaraStart") index=SCI-EXPANDED, SSCI	
#3	TS=(sensitivity or specificiy or (detection SAME rate*) or (predictive SAME value*) or (reference SAME value*) or performance or (screen SAME positive*) or accura* or reliab* or "false postive result*" or "false negative result*" or "diagnostic accuracy" or "diagnostic error" or "laboratory diagnosis" or "false positive*" or "false negative*") index=SCI-EXPANDED, SSCI	
#4	#1 AND #2 AND #3 index=SCI-EXPANDED, SSCI	2698
	EMBASE	
#1	'malaria'/exp	
#2	'plasmodium'/exp	
#3	'plasmodium' OR 'malaria':ab,kw,ti	
#4	#1 OR #2 OR #3	
#5	rdt:ab,kw,ti	
#6	'rapid diagnostic test'/exp	
#7	'rapid diagnos* test*' OR Dipstick* OR 'Rapid diagnos* device*':ab,kw,ti	
#8	'immunoaffinity chromatography'/exp	
#9	'immunochromatograph*':ab,kw,ti	
#10	'antigen detection method*':ab,kw,ti	
#11	'rapid malaria antigen test*':ab,kw,ti	
#12	'chromatography'/exp	
#13	'chromatography':ab,kw,ti	
#14	'enzyme linked immunosorbent assay'/exp	
#15	'enzyme linked immunosorbent assay':ab,kw,ti	
#16	'rapid test'/exp	
#17	'rapid test':ab,kw,ti	
#18	'reagent strip*':ab,kw,ti	
#19	'agglutination test'/exp	
#20	'agglutination test':ab,kw,ti	
#21	'immunoassay'/exp	
#22	immunoassay* OR 'immuno assay*':ab,kw,ti	
#23	'OptiMal®' OR 'ParaSight-F' OR 'Binax NOW®' OR 'ICT Malaria' OR 'CaraStart':ab,kw,ti	
#24	#5 OR #6 OR #7 OR #8 OR #9 OR #10 OR #11 OR #12 OR #13 OR #14 OR #15 OR #16 OR #17 OR #18 OR #19 OR #20 OR #21 OR #22 OR #23	
#25	'sensitivity and specificity'/exp	
#26	'sensitivity analysis'/exp	
#27	'reference value'/exp	
#28	'diagnostic accuracy'/exp	
#29	'false positive result'/exp	
#30	'false negative result'/exp	

#31	'laboratory diagnosis'/exp	
#32	'diagnostic error'/exp	
#33	sensitivity:ab,kw,ti	
#34	specificity:ab,kw,ti	
#35	'reference value':ab,kw,ti	
#36	'false positive*':ab,kw,ti	
#37	'false negative*':ab,kw,ti	
#38	'detection rate':ab,kw,ti	
#39	'laboratory diagnosis':ab,kw,ti	
#40	'diagnostic accuracy':ab,kw,ti	
#41	'diagnostic error':ab,kw,ti	
#42	#25 OR #26 OR #27 OR #28 OR #29 OR #30 OR #31 OR #32 OR #33 OR #34 OR #35 OR #36 OR #37 OR #38 OR #39 OR #40 OR #41	
#43	performance'/exp	
#44	performance':ab,kw,ti	
#45	#42 OR #43 OR #44	
#46	#4 AND #24 AND #45	3284
	Cochrane Library	
#1	MeSH descriptor: [Malaria] explode all trees	
#2	(plasmodium):ti,ab,kw OR (malaria):ti,ab,kw	
#3	#1 OR #2	
#4	MeSH descriptor: [Chromatography] explode all trees	
#5	(RDT):ti,ab,kw OR ('rapid diagnos* test*'):ti,ab,kw OR (dipstick*):ti,ab,kw OR ('rapid diagnos* device*'):ti,ab,kw	
#6	MeSH descriptor: [Immunochromatography] explode all trees	
#7	(immunochromatograph*):ti,ab,kw OR ('chromatography'):ti,ab,kw OR ('antigen detection method*'):ti,ab,kw OR ('rapidmalaria antigen test*'):ti,ab,kw	
#8	MeSH descriptor: [Immunoassay] explode all trees	
#9	('enzyme linked immunosorbent assay'):ti,ab,kw OR ('rapid test'):ti,ab,kw OR ('reagent strip*'):ti,ab,kw OR ('agglutination test'):ti,ab,kw OR ('immunoassay'):ti,ab,kw	
#10	('OptiMal®'):ti,ab,kw OR ('ParaSight-F'):ti,ab,kw OR ('Binax NOW®'):ti,ab,kw OR ('ICT Malaria'):ti,ab,kw OR ('CaraStart'):ti,ab,kw	
#11	#4 OR #5 OR #6 OR #7 OR #8 OR #9 OR #10	
#12	MeSH descriptor: [Sensitivity and Specificity] explode all trees	
#13	(sensitivity or specificity or 'reference value*' or performance or 'screen positive*' or 'screen accura*' or 'screen reliab*') (Word variations have been searched)	
#14	MeSH descriptor: [Reference Values] explode all trees	
#15	("false postive result*" or "false negative result*" or "diagnostic accuracy" or "diagnostic error" or "laboratory diagnosis" or "false positive*" or "false negative*") (Word variations have been searched)	
#16	#12 OR #13 OR #14 OR #15	
#17	#3 AND #11 AND #16	210
	the China National Knowledge Infrastructure	

#1	(SU = '疟疾' OR SU = '疟原虫') AND (SU = 'RDT' OR SU = '免疫层析' OR SU = '快速免疫诊断' OR SU = 'OptiMal' OR SU = 'ParaSight-F' OR SU = 'Binax NOW' OR SU = 'ICT Malaria' OR SU = 'CaraStart'OR SU = '快速检测' OR SU = '快速诊断')	192
	Wanfang Data	
#1	(主题:"疟疾"+主题:"疟原虫")*(主题:"RDT"+主题:"免疫层析"+主题:"快速免疫诊断"+主题:"OptiMal®"+主题:"ParaSight-F"+主题:"Binax NOW"+主题:"ICT Malaria"+主题:"CaraStart"+主题:"快速检测"+主题:"快速诊断")*Date:-2018	573
	Sinomed	
#1	(((("疟疾"[常用字段]) OR "疟疾"[不加权:扩展]) OR "疟原虫"[常用字段]) OR "疟原虫"[不加权:扩展])AND (((((((("RDT"[常用字段]) OR "免疫层析"[常用字段]) OR "快速免疫诊断"[常用字段]) OR "快速检测"[常用字段]) OR "快速诊断"[常用字段]) OR "OptiMal"[常用字段]) OR "ParaSight-F"[常用字段]) OR "Binax NOW"[常用字段]) OR "ICT Malaria"[常用字段]	218

Appendix S3: QUADAS-2 checklist for methodological quality assessment of included studies

Patient Selection	
**A. Risk of Bias**	
Was a consecutive or random sample of patients enrolled?	• ’Yes’ if the sampling method is consecutive or random, or if all patients screened who are eligible are recruited.<br>• ’No’ if the sampling method is convenience.<br>• ’Unclear’ if the sampling method is not clearly described.
Was a case-control design avoided?	• ’Yes’ if the study is not a case-control design.<br>• ’No’ if the study is a case-control design.<br>• ’Unclear’ if the study design is not clearly described.
Did the study avoid inappropriate exclusions?	• ’Yes’ if exclusion criteria are clearly listed and appropriate<br>• ’No’ if exclusion criteria are clearly listed but inappropriate<br>• ’Unclear’ if exclusion criteria are not listed.
Could the selection of patients have introduced bias?	• ’Low’ if the answer to all signalling questions is 'Yes'.<br>• ’High’ if the answer to either of the signalling questions is 'No'.<br>• ’Unclear’ if the answer to at least one signalling question is "Unclear" and neither is 'No'.
**B. Concerns regarding applicability**	
Are there concerns that the included patients and setting do not match the review question?	• ’Low’ if the patients included are symptomatic children or teenagers<br>• ’High’ if the sample is not representative of people with malaria parasitaemia in general. (eg.the study only focuses on cerebral malaria or asymptomatic subjects)<br>• ’Unclear’ if the study design or the sampling method is not well described.
**All tests**	
**A. Risk of Bias**	
Were the index test results interpreted without knowledge of the results of the reference standard?	• ’Yes’ if unawareness of the person undertaking the index test of the results of the reference test is explicitly mentioned, or the index test was undertaken before the reference test.<br>• ’No’ if the person undertaking the index test was aware of the results of the reference test.<br>• ’Unclear’ if the blinding status is not mentioned or not clearly described.
Could the conduct or interpretation of the index test have introduced bias?	• ’Low’ if the answer to signalling question is 'Yes'.<br>• ’High’ if the answer to signalling question is 'No'.<br>• ’Unclear’ if the answer to signalling question is "Unclear".
**B. Concerns regarding applicability**	
Are there concerns that the index test, its conduct, or interpretation differ from the review question?	• ’Low’ if the index test was performed in field conditions.<br>• ’High’ if the index test was not performed in field conditions(eg.in lab).<br>• ’Unclear’ if it is not clearly described.
**Reference Standard**	
**A. Risk of Bias**	
Is the reference standards likely to correctly classify the target condition?	• ’Yes’ if expert microscopic examination of thick and thin blood smears is explicitly mentioned and if more than one microscopists viewed slides.<br>• ’Yes’ if reference standard was PCR.<br>• ’No’ if there is only one microscopist involved, or if only thick blood smears were examined.<br>• ’Unclear’ if sufficient information is not provided.

Were the reference standard results interpreted without knowledge of the results of the index tests?	• 'Yes' if unawareness of the person undertaking the reference test of the results of the index test is explicitly mentioned, or the reference test was undertaken before the index test.<br>• 'No' if the person undertaking the reference test was aware of the results of the index test.<br>• 'Unclear' if the blinding status is not mentioned or not clearly described.
Could the reference standard, its conduct, or its interpretation have introduced bias?	• 'Low' if the answer to both signalling questions is 'Yes'.<br>• 'High' if the answer to either of the signalling questions is 'No'.<br>• 'Unclear' if the answer to at least one signalling question is "Unclear" and neither is 'No'.
**B. Concerns regarding applicability**	
Are there concerns that the target condition as defined by the reference standard does not match the question?	• 'Low' if the target condition matches the question.<br>• 'High' if the target condition doesn't match the question.<br>• 'Unclear' if the target condition is not clear.
**Flow and Timing**	
**A. Risk of Bias**	
Was there an appropriate interval between index test and reference standard?	• 'Yes' if both tests were undertaken on the same day of sample collection or after storage at an appropriate temperature.<br>• 'No' if any of the tests were undertaken on a different day<br>• 'Unclear' if the timing of the tests is not clearly described.
Did all patients receive a reference standard?	•'Yes' if all participants who received the index test also received the reference test.<br>•'No' if not all the participants who received the index test also received the reference test.<br>•'Unclear' if insufficient information was provided to assess this.
Did all patients receive the same reference standard?	• 'Yes' if it is clear that all patients received the same reference standard.<br>• 'No' if subgroups of patients received different reference standards.<br>• 'Unclear' if the information cannot be obtained.
Were all patients included in the analysis?	• 'Yes' if the number of patients included equals to the number of patients used in the analysis.<br>• 'No' if the number of patients included does not equal to the number of patients used in the analysis and no appropriate reason is provided for this.<br>• 'Unclear' if the information is not clear.
Could the patient flow have introduced bias?	• 'Low' if the answer to all signalling questions is 'Yes'.<br>• 'High' if the answer to either of the signalling questions is 'No'.<br>• 'Unclear' if the answer to at least one signalling question is "Unclear" and neither is 'No'.

Appendix S4: PRISMA checklist

Section/topic	#	Checklist item	Reported on page #
**TITLE**			
Title	1	Identify the report as a systematic review, meta-analysis, or both.	1
**ABSTRACT**			
Structured summary	2	Provide a structured summary including, as applicable: background; objectives; data sources; study eligibility criteria, participants, and interventions; study appraisal and synthesis methods; results; limitations; conclusions and implications of key findings; systematic review registration number.	1
**INTRODUCTION**			
Rationale	3	Describe the rationale for the review in the context of what is already known.	2-3
Objectives	4	Provide an explicit statement of questions being addressed with reference to participants, interventions, comparisons, outcomes, and study design (PICOS).	3
**METHODS**			
Protocol and registration	5	Indicate if a review protocol exists, if and where it can be accessed (e.g., Web address), and, if available, provide registration information including registration number.	NA
Eligibility criteria	6	Specify study characteristics (e.g., PICOS, length of follow-up) and report characteristics (e.g., years considered, language, publication status) used as criteria for eligibility, giving rationale.	3
Information sources	7	Describe all information sources (e.g., databases with dates of coverage, contact with study authors to identify additional studies) in the search and date last searched.	3
Search	8	Present full electronic search strategy for at least one database, including any limits used, such that it could be repeated.	appendix 2
Study selection	9	State the process for selecting studies (i.e., screening, eligibility, included in systematic review, and, if applicable, included in the meta-analysis).	3
Data collection process	10	Describe method of data extraction from reports (e.g., piloted forms, independently, in duplicate) and any processes for obtaining and confirming data from investigators.	3
Data items	11	List and define all variables for which data were sought (e.g., PICOS, funding sources) and any assumptions and simplifications made.	3
Risk of bias in individual studies	12	Describe methods used for assessing risk of bias of individual studies (including specification of whether this was done at the study or outcome level), and how this information is to be used in any data synthesis.	3
Summary measures	13	State the principal summary measures (e.g., risk ratio, difference in means).	4
Synthesis of results	14	Describe the methods of handling data and combining results of studies, if done, including measures of consistency (e.g., I^2) for each meta-analysis.	4
Risk of bias across studies	15	Specify any assessment of risk of bias that may affect the cumulative evidence (e.g., publication bias, selective reporting within studies).	4
Additional analyses	16	Describe methods of additional analyses (e.g., sensitivity or subgroup analyses, meta-regression), if done, indicating which were pre-specified.	4
**RESULTS**			

Study selection	17	Give numbers of studies screened, assessed for eligibility, and included in the review, with reasons for exclusions at each stage, ideally with a flow diagram.	4
Study characteristics	18	For each study, present characteristics for which data were extracted (e.g., study size, PICOS, follow-up period) and provide the citations.	4-5, 7-8
Risk of bias within studies	19	Present data on risk of bias of each study and, if available, any outcome level assessment (see item 12).	5
Results of individual studies	20	For all outcomes considered (benefits or harms), present, for each study: (a) simple summary data for each intervention group (b) effect estimates and confidence intervals, ideally with a forest plot.	5-6
Synthesis of results	21	Present results of each meta-analysis done, including confidence intervals and measures of consistency.	5
Risk of bias across studies	22	Present results of any assessment of risk of bias across studies (see Item 15).	5
Additional analysis	23	Give results of additional analyses, if done (e.g., sensitivity or subgroup analyses, meta-regression [see Item 16]).	5
**DISCUSSION**			
Summary of evidence	24	Summarize the main findings including the strength of evidence for each main outcome; consider their relevance to key groups (e.g., healthcare providers, users, and policy makers).	11
Limitations	25	Discuss limitations at study and outcome level (e.g., risk of bias), and at review-level (e.g., incomplete retrieval of identified research, reporting bias).	11
Conclusions	26	Provide a general interpretation of the results in the context of other evidence, and implications for future research.	11-12
**FUNDING**			
Funding	27	Describe sources of funding for the systematic review and other support (e.g., supply of data); role of funders for the systematic review.	12
